# Physiology

**Published:** 1878-09

**Authors:** 


*Collaborators:*

Dr. H. Gradle, Dr. L. W. Case, Dr. R. Park,
Dr. R. Tilley, Dr. D. R. Brower.

### Physiology.

### Experimental Investigations on the Variations of the Volume of the Cranium, and on the Application of the Graphic Method to Solve the different Anthropological Problems.—M. Le Bon. (*La France Médicale*, July 17, 1878.)

M. B. presented to *L'Académie des Sciences* the following conclusions which are based on a considerable number of measurements, both of living subjects and from the specimens in the Museum of Anthropology, as well as from unpublished documents. The results are exhibited in graphic form at the Exposition. The conclusions are as follows:

1. The development of intelligence bears an intimate relation with the form, structure and volume of the brain. The volume is one of the most important of these factors. In measuring a series of skulls, taking a good average number to form the series, it invariably holds good that the most voluminous brain belongs, in the human species, to the races the most gifted intellectually; and in each race to the most intelligent individual.

2. In confining the attention, as is generally done, to the mean of all the skulls of each race and by comparing these means, there appears to be very little difference between one race and another. But if with the measurements arranged according to their increasing volume, certain curved lines are drawn exhibiting how many subjects there are in a given race possessing a brain of given volume, it is readily seen that that which constitutes the superiority of one race over another is the fact that the superior race contains many more large skulls than the inferior race. In a hundred modern Parisian skulls there are about eleven subjects the volume of whose skulls would range between 1,700 and 1,900 cubic centimeters, whilst among the same number of negroes not one is found with a capacity equal to those indicated above.

The weight of 100 brains of modern Parisians of the masculine sex has shown that their weight varies from 1,000 to 1,700 grams. The cubic measure of an equal number of skulls has demonstrated that the measure of an individual skull varies from 1,300 to 1,900 cubic centimeters. These extremes approach each other by gradual progression.

4. The remarkable difference which has just been indicated in the weight of the brain and size of skull of different individuals of the same race, varies remarkably when we compare one race with another. The difference is greater in proportion as the race is more advanced in the scale of civilization. It has been established that the difference between the volume of the largest adult masculine skulls and the smaller skulls among the following races are :

| | | |
|---|---|---|
| Difference of size of the Gorrilla skull.............. | 148 | C. C. |
| Difference of size of the Negro skull ............... | 204 | " |
| Difference of size of the Ancient Egyptian ........ | 353 | " |
| Difference of size of the Parisian of XVII century | 472 | " |
| Difference of size of the modern Parisian .......... | 593 | " |

The difference in size between the greatest and smallest skulls of modern Parisians is three times as great as the same difference among the negroes. The difference is greater among the modern Parisians than among their ancestors of 600 years ago.

5. The height (*taille*) has a certain amount of influence over the size of the brain, but the influence is very small. In grouping individuals of the same height (*taille*) and taking the mean weight of the brain of each group, it is perceived that between the mean weight of the brain of the group of individuals of the greatest height and the mean weight of the brain of the individuals of the smallest height, the difference is scarcely 100 grams, whilst the difference often amounts to 300 grams among individuals of the same height.

6. Similar height being compared, the female has a brain much lighter than the male. On taking the mean weight of 17 brains of the masculine sex, the height being from 159 to 160 centimeters and comparing them with the brains of 17 of the female sex, the difference was found to be 172 grams in favor of the males.

7. The difference existing between the weight of the brain and consequently the volume of the skull in man and woman increases constantly in proportion as the race advances in the scale of civilization; so that looking at the mass of the brain and consequently the intelligence, the difference which was previously stated as existing is still increasing. For instance, the difference which exists now between the mean size of the skulls of the men of Paris and the women of Paris, is almost double that which exists between the skulls of the male and female inhabitants of Egypt.

8. Certain persons, having skulls measuring the same circumference, may present a difference of height of skull amounting to 200 C. C. in volume. That is easily comprehended when we remember the different factors, especially the height of skull, which must be taken into consideration in order to estimate the volume. But in measuring a whole series it is soon demonstrated that an increase of one centimeter in circumference of the skull corresponds to an increase of volume oscillating about 100 C. C.

9. The circumference of the skull, on which depends, as has just been seen, the volume of the brain, bears an intimate relation with the intelligence.

10. A comparative study of the various curves of the circumference of the skull, and of the curves of the head, and of the volume and weight of the brain, has shown the relation existing between these different values. A head, with a circumference of 57 centimeters, corresponds to a skull of 52 centimeters, and to a volume of 1,550 C. C. The probable weight of the brain in the skull will be 1,250 grams.

11. There is a constant inequality of development between the two halves of the skull which is sometimes more developed on the left, sometimes on the right side. Neither the race nor the intelligence seems to have any perceptible influence on this unequal development.

